# The protective role of statins in COVID-19 patients: a retrospective observational study

**DOI:** 10.1186/s41231-021-00102-4

**Published:** 2021-09-25

**Authors:** Srikanth Umakanthan, Sanjum Senthil, Stanley John, Mahesh K. Madhavan, Jessica Das, Sonal Patil, Ragunath Rameshwaram, Ananya Cintham, Venkatesh Subramaniam, Madhusudan Yogi, Abhishek Bansal, Sumesh Achutham, Chandini Shekar, Vijay Murthy, Robbin Selvaraj

**Affiliations:** 1 Department of Para-clinical sciences, Faculty of Medical Sciences, The University of the West Indies, St Augustine, Trinidad Trinidad and Tobago; 2 International Research Association Unit, New Delhi, India; 3 Department of Medicine, RRN Multispecialty Hospital, Tamil Nadu, India; 4 Department of Medicine, Holy Cross Hospital, Tamil Nadu, India; 5 National Regional Collaboration for Medical Research Foundation, New Delhi, India; 6 Department of Biostatistics, Epidemiology, and Informatics, Piramal Research Centre, Gujarat, India; 7 Swaminathan Multispecialty Hospital, Chennai, India

**Keywords:** COVID-19, Comorbidities, Confounders, Outcome

## Abstract

**Background:**

To evaluate and determine the protective role of statins in COVID-19 patients.

**Methods:**

This is a retrospective cohort study conducted across five hospitals in India. Patients diagnosed with COVID-19 and hospitalized with existing and valid medical documentation were included.

**Results:**

This study comprised 3252 COVID-19 patients, of whom 1048 (32.2%) were on statins, with 52.4% being males. The comorbidity prevalence of hypertension was 75%, followed by diabetes 62.51% and coronary artery disease being 47.5%. At the time of hospitalization, statin users had a higher incidence of dyspnea, cough, and fatigue (95.8, 93.3, and 92.7%). The laboratory results revealed a lower mean of WBC count (7.8 × 10^3^/μL), D-dimer (2.4 μg/mL), and C-reactive protein (103 mg/L) among statin users. They also had lower mortality rates (17.1%), a lesser requirement for mechanical ventilation (20%), and hemodialysis (5.4%).

**Conclusion:**

This observation study elaborates on the beneficial effects of statins in COVID-19 patients. However, the inferences from this study should be viewed with caution due to the impending effect of confounding factors on its statistical results.

## Background

The novel SARS-CoV-2 infection initiated a worldwide pandemic in March 2021 [1]. The patients with coronavirus disease (COVID-19) were hospitalized with varying systemic signs and symptoms ranging from dyspnea to acute respiratory distress syndrome (ARDS) requiring mechanical ventilation [2]. The clinical manifestations exhibited by the COVID-19 patients, along with the impending complications, the management of COVID-19, vary from symptomatic treatment to mechanical ventilation [3]. The initial treatment criteria rely on spirometry, computerized tomography (CT) scan, and endothelial function [4, 5]. Systemic complications of COVID-19 include arrhythmias, coagulopathies, acute liver injury, septic shock, and multi-organ failure. COVID-19 is more commonly seen in individuals with existing comorbidities such as chronic lung disease, liver disease, overweight and obesity, diabetes mellitus, and hypertension [6]. In China, a study done by Guan et al., concluded that the severity of COVID-19 was more in patients with pre-existing diseases such as diabetes mellitus, ischemic heart disease and systemic hypertension [7]. Statins are a group of drugs that lower the low-density lipoprotein (LDL) cholesterol by their anti-inflammatory and antithrombotic action. The collective action of statins provides a protective effect in patients with ischemic heart disease, atherosclerosis, and in patients experiencing vascular complications, as shown in a retrospective study conducted in China [8–10]. SARS-CoV-2 gains entry through the host ACE2 receptors expressed by type II alveolar epithelial cells in the lung. Statin’s augment ACE2 expression and reduce the inflammatory activity within the pulmonary vasculature, improving the clinical outcome and reducing mortality in COVID-19 patients [11, 12]. The molecular mechanism of action for statins includes decreased in C-reactive protein levels, inhibition of miR-133a expression, interference with Kruppel-like factor 2 signaling, and modulating high mobility group box 1/ toll-like receptor 4(HMGB1/TLR4) pathway [13]. Statins exhibit immunomodulatory actions in several autoimmune diseases (lupus erythematosus, rheumatoid arthritis, and ankylosing spondylitis) [14]. These immunomodulatory effects by statins are produced by reducing the major histocompatibility complex (MHC) class II expression, resulting in suppression of antigen presentation and T-cell activation [15]. The other beneficial effects of statins are their anti-inflammatory effects in cardiac surgery and improving lung compliance in chronic lung diseases, pneumonia, and ARDS [16]. The innumerable beneficial effects of statins are widely explored in medicine; however, this wonder drug is associated with few adverse effects, as reported in previous literature studies. The most common adverse effects reported are direct self-limited myotoxicity, diabetes mellitus, followed by other rarer adverse effects such as elevated serum liver enzymes, memory loss, and risk of developing cataracts [9]. Few observational studies have claimed statins are associated with neuropathy, sleep disorders, and erectile dysfunction [17, 18].

In observance of the beneficial effects of statins in combatting COVID-19 infection through their antagonist effects on the SARS-CoV-2, we conducted an observational study to scrutinize the agonist effects of statins in hospitalized COVID-19 patients [19]. Thus far, numerous studies have analyzed the beneficial effects of statins in treating viral pneumonia and ARDS. Our present study evaluates the patients’ demographics, persisting comorbidities, clinical manifestations, and variations among unmatched and matched cohort pools.

## Methods

### Data sources and study population

The Indian citizens are provided with a personal identity number based on their demographic data and biometrics [20]. Our study performed data collection across five hospitals using a personal coding sequence, identifying the patient’s age, gender, chief medical complaints, relevant investigation reports and treatment history. The personal coding sequence allowed us to de-identify the patients and maintain a high confidentiality level for a secured data storage.

Our study included hospitalized COVID-19 patients across five hospitals in India during the pandemic period ranging from 1st June 2020 until 31th May 2021 with confirmed COVID-19 by RT-PCR test. The samples collected from these patients were from either oropharynx or nasopharynx. The RT-PCR test results were supported by imaging studies (computerized tomography of the chest) and low oxygen saturation (SPO2) of ≤93%.

Patients with a lack of medical documentation, suffering from chronic bacterial and non-COVID-19 viral infections, malignancies, and receiving radiotherapies were excluded from the study. A total of 3252 patients were selected for our study. The joint board of research ethics provided ethics approval and granted waiver for informed consent. Initial case finding tally of 3668 patients were identified and reviewed based on the inclusion criteria. Of these patients, following scrutiny, a total of 3252 patients were identified for our study.

The in-hospital data search was conducted using a digital database search engine provided in the sections of the medical records in all five hospitals. Manual extraction was prohibited due to the ongoing COVID-19 restrictions and the data search involved identifying patients age, gender, BMI, chief presenting signs and symptoms. The selected patients were further checked for laboratory test results and treatment history. The treatment details were identified and followed across the duration of hospital stay with an emphasis on statin therapy. The search concluded with the patient’s primary outcome (mortality) or the secondary outcome (involved mechanical ventilation). The selected patient’s data was given an alphanumerical coding sequence to deidentify the patient.

In our study cohort, 1048 (32.2%) were statin users, and the remaining 2204 (67.7%) were non-statin users. Among 1536 patients, a 1:1 propensity-score matching (768 statin users and 768 non-statin users) was performed. The differences between statin and non-statin users were further evaluated based on their age, gender, BMI, clinical manifestations, period of COVID-19 disease, duration of statin intake, and presence of comorbid conditions. Chief investigator and consultants reviewed the collected data and inaccurate data were further searched for accurate information.

### Statistical analyses

In our study, we investigated the association of statin use with patient’s age, gender, BMI, pre-existing comorbidities, commonly associated clinical signs, and symptoms and finally, the prognostic outcome of the patients. The statistical evaluation was noted as total number percentage [n(%)] for nominal variables, medians, and inter-quantile ranges (IQR) for measuring variables. Statistical evaluation was performed by using univariable logistic regression to screen for predictors [21]. This was followed by multivariable logistic regression and propensity matching to statistically analyze and compare the general characteristics and most frequently occurring clinical manifestation of COVID-19 patients at the time of hospitalization [22]. Laboratory results and patient’s prognostic outcomes were stratified based on statin use in the cohort group of hospitalized COVID-19 patients.

**Fig. 1:**
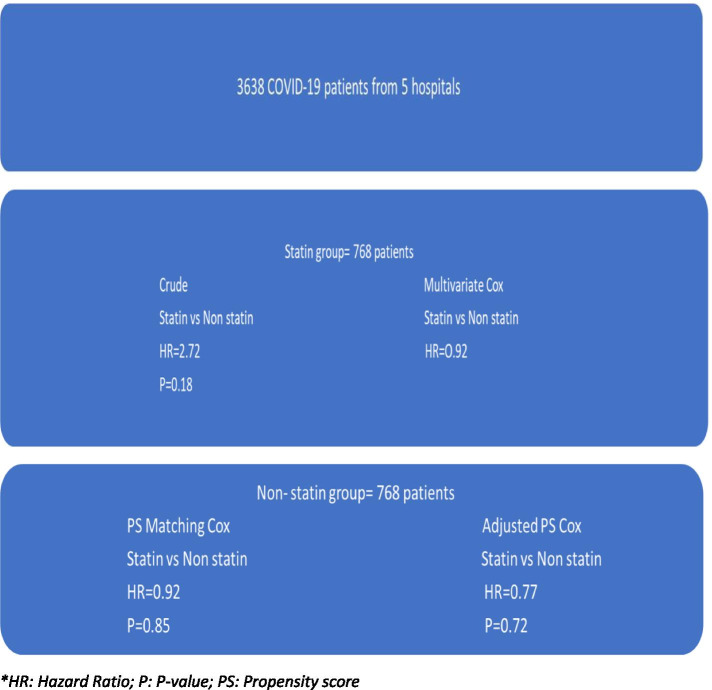
Cox proportional-hazards regression scores in the cohort study pools. *HR: Hazard Ratio; P: *P*-value; PS: Propensity score

Cox regression models were used to deliberate between statin and non-statin users [23]. The Cox regression model probes the association of the predictors with the time-to-event model through its hazard function. Since our study is associated with numerous predictors in the form of confounding factors, these factors can generate statistical variance and errors in the results, creating significant biased outcomes in observational studies. To avoid this statistical bias, the Cox proportional-hazard regression model was implemented to evaluate the relationship between statin use and the clinical outcome of the COVID-19 hospitalized patients [24]. The propensity-hazards in Cox regression models avoided time-varying predictors during our statistical analyses [25]. This was followed by univariate and multivariate regression tests depending on single or multiple variables at the test. The confounding effects were further quantified into crude, multivariate, propensity score matching and adjusted propensity score categories. The confounders in our study included clinical features, laboratory results, and drugs (antibiotics and glucocorticoids). Since our study was a cohort study, the propensity of confounding factors could generate variance, bias, and result in extensive statistical errors [26]. This can be avoided by rigorous randomization of participants based on treatment strategies (Randomized control trial). However, in our cohort study, the hazards of confounding factors were minimized by introducing propensity scores [27]. The unique propensity was estimated using a multivariable logistic regression model (Fig. 1). Statistical package for social science (SPSS) software was used for statistical analyses [28].

## Results

In a cohort pool of 3252 participants with COVID-19, 1048 (32.2%) were statin users. The mean age for statin users was 63 years (with IQR of 55-79 years) compared to 59 years (with IQR of 45-77 years) among non-statin COVID-19 patients. Males (55.84%) were more affected compared to females (44.15%) in the statin user pool with *p* = 0.05.

Statin users were more commonly associated with comorbidities, including hypertension (75.0% vs 58.0%), diabetes mellitus (62.5% vs 40.3%), coronary artery disease (47.5% vs 37.1%), heart failure (41.9% vs 33.0%), chronic lung disease (39.1% vs 24.3%), chronic kidney disease (37.2% vs 15.9%), cerebrovascular accidents (11.0% vs 2.9%) [*p* < 0.01 for both], and liver disease (6.8% vs 3.9%) (Table 1).

**Table 1:** Hospitalized patients demographics, persisting comorbidities, clinical manifestations, and variations among unmatched and matched cohort pool

**Total* N* = 3252**	**Unmatched**			**Matched**		
	**Statin use 1048 (32.2%)**	**No statin use 2204 (67.7%)**	***p* value**	**Statin use (*n* = 768)**	**No statin use (*n* = 768)**	***p* value**
**Demographics**						
Age (years)	63 (55–79)	59 (45–77)	< 0.001	62 (54–76)	64 (55–77)	0.16
Gender			0.05			1.0
*Male*	550 (52.4%)	1266 (57.4%)		422 (54.9%)	446 (58.0%)	
*Female*	498 (47.5%)	938 (42.5%)		346 (45.0%)	322 (41.9%)	
BMI (kg/m^2^)	28.2 (24.1–31.8)	27.8(24.4–32.0)	0.24	28.3 (24.6–32.4)	27.2 (23.8–31.8)	0.62
Comorbidities						
*HTN*	786 (75.0%)	1280 (58.0%)	< 0.001	484 (63.0%)	546 (71.0%)	0.45
*DM*	656 (62.5%)	890 (40.3%)	< 0.001	424 (55.2%)	448 (58.3%)	0.54
*CAD*	498 (47.5%)	818 (37.1%)	< 0.001	124 (16.1%)	116 (15.1%)	0.74
*CLD*	410 (39.1%)	536 (24.3%)	< 0.001	186 (24.2%)	192 (25.0%)	0.45
*CKD*	390 (37.2%)	352 (15.9%)	< 0.01	168 (21.8%)	160 (20.8%)	0.75
*CVA*	116 (11%)	66 (2.9%)	< 0.01	70 (9.1%)	66 (8.5%)	0.86
*Heart failure*	440 (41.9%)	728 (33.0%)	0.96	128 (16.6%)	124 (16.1%)	0.88
*Liver disease*	72 (6.8%)	88 (3.9%)	0.98	72 (9.3%)	56 (7.2%)	0.70
**Clinical features**						
Fever (^o^c)	37.45 ± 1.16	37.41 ± 1.04	0.787	37.31 ± 1.09	37.22 ± 1.04	0.60
Dyspnoea n*(%)*	1004 (95.8%)	1720 (78.0%)	< 0.01	640 (83.3%)	624 (81.2%)	0.88
Cough *n (%)*	978 (93.3%)	1802 (81.7%)	< 0.01	606 (78.9%)	594 (77.3%)	0.87
Chest pain *n(%)*	52 (4.9%)	108 (4.9%)	< 0.001	70 (9.1%)	78 (10.1%)	0.88
Fatigue *n (%)*	972 (92.7%)	1686 (76.4%)	< 0.01	592 (77.0%)	602 (78.3%)	0.86
O_2_ saturation (Spo_2_ ≤ 93%)	314 (29.9%)	1328 (30.1%)	< 0.001	228 (29.6%)	216 (28.1%)	0.88

*BMI* Body mass index, *HTN* Hypertension, *DM* Diabetes mellitus, *CAD* Coronary artery disease, *CLD* Chronic lung disease, *CKD* Chronic kidney disease, *CVA* Cerebrovascular accident

The statistical estimates focusing on the most frequently occurring clinical manifestation revealed that patients on statins had a high incidence of dyspnea, cough, and fatigue (95.8, 93.3, and 92.7%, respectively) in comparison to non-statin patients (78.0, 81.7, and 76.4% respectively. There were few differences in patient presentation incidence with chest pain and low o2 saturation among statin and non-statin users (Table 1).

To evaluate the cohort incidence among the relevant laboratory findings, a 1:1 propensity-matched cohort was performed. The hospitalized COVID-19 patients with statins showed a lower mean of C-reactive protein (103 mg/L vs. 124.7 mg/L, *p* < 0.001) D-dimer (2.4 μg/mL vs. 2.8 μg/mL, p 0.37), and WBC count (7.8 × 10^3^/μL vs. 8.5 × 10^3^/μL, *p* < 0.01), in comparison to non-statin users. The same positive results followed in lipid profiles for patients on statins (Table 2). Patients with antecedent statin use had lesser requirement for mechanical ventilation (20% vs. 24.2%, p 0.07), hemodialysis (5.4% vs. 7%, p 0.41) and showed lower mortality rates (17.1% vs. 31%, *p* < 0.001) compared to non-statin patients. There were no significant differences in length of hospital stay, antibiotic administration, and days on a ventilator (Table 3).

**Table 2:** Laboratory indices in matched cohort study pool

**Laboratory indices**	**Matched hospitalized COVID-19 patients**		
	**Statin users (*n* = 768)**	**No statin users (*n* = 768)**	***p* value**
*CRP (mg/ml)*	103.0 (48.1–172.2)	124.7 (72.2–196.6)	< 0.001
*ESR (mm/hr)*	67.5 (36.3–98.1)	66.5 (34.4–93.6)	0.84
*WBC count (10*^*3*^*/μL)*	7.8 (5.3–11.2)	8.5 (5.4–11.8)	< 0.01
*D-dimer (μg/ml)*	2.4 (1.2–4.0)	2.8 (1.2–4.8)	0.37
*Total cholesterol (mg/dL)*	154.2 (118.3–186.0)	164.6 (133.0–203.7)	< 0.01
*Triglycerides (mg/dL)*	144.0 (99.2–192.3)	138.0 (94.7–216.7)	0.25
*HDL (mg/dL)*	40.0 (32.2–53.3)	44.0 (34.4–56.4)	0.25
*LDL (mg/dL)*	79.1 (58.0–110.2)	92.0 (68.0–115.0)	< 0.01

*WBC* White blood cells, *CRP* C-reactive protein, *ESR* Erythrocyte sedimentation rates, *LDL* low density lipoprotein, *HDL* High density lipoprotein

**Table 3:** COVID-19 hospitalized patient’s outcome and clinical interventions in matched cohort study pool

**Clinical Variable**	**Statin use (*n* = 768)**	**No statin use (*n* = 768)**	***P* value**
Hemodialysis *n (%)*	42 (5.4%)	54 (7.0%)	0.41
Days on ventilator	13.5 (3.2–31.0)	12.8 (2.0–34.1)	0.77
Length of hospital stay *(days)*	7.0 (3.0–12.0)	7.0 (4.0–1.0)	0.27
Mortality after hospitalization *n (%)*	132 (17.1%)	238 (31%)	< 0.001

**Fig. 2:**
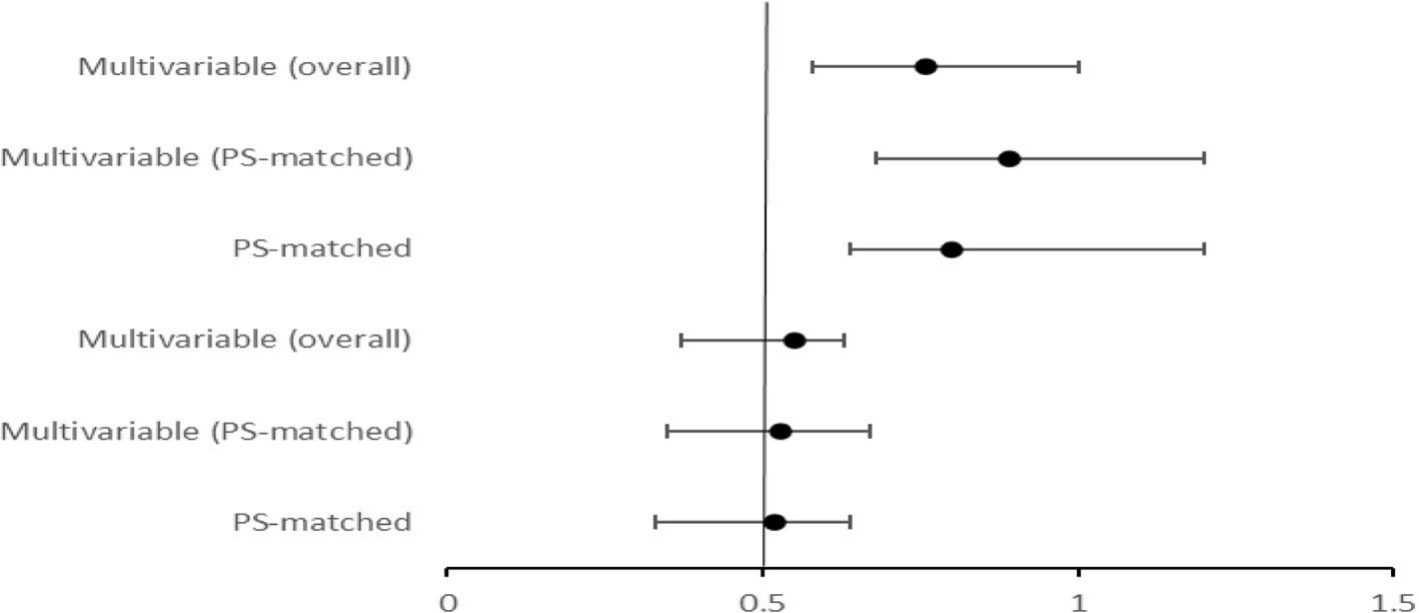
Forest plot demonstrating the odds ratio and 95% confidence interval for in-hospital mortality with statin use depicting primary endpoint and secondary endpoints

The clinical outcome among hospitalized COVID-19 statin users was evaluated using multivariable adjusted cohort and propensity matched cohort. Odds ratio (OR), 95% confidence interval (CI) was evaluated for primary and secondary endpoint. Statin users had a significant favorable clinical outcome with a lower mortality compared to non-statin users. The primary endpoint estimated the mortality rates during hospitalization whereas the secondary endpoint included patients that required mechanical ventilation. The overall multivariable primary odds ratio was 0.55 and overall multivariable secondary odds ratio was 0.76. The multivariable (PS-matched) OR and 95% CI in primary endpoint hospitalized patients was 0.53 and 0.35-0.67. The multivariable (PS-matched) OR and 95% CI in secondary endpoint hospitalized patients was 0.89 and 0.68-1.20 (Fig. 2).

## Discussion

The effect of statins on COVID-19 patients has been debatable based on numerous literature studies [29–31]. Our study involved a high number of COVID-19 participants, and the significant findings in our evaluation show that antecedent statin users were older males with high BMI, had a frequent association of pre-existing comorbidities including diabetes mellitus, coronary artery disease, hypertension, heart failure, chronic lung disease, and chronic kidney disease. The laboratory parameters were more favorable, and the mortality was significantly lower among statin users.

Our study showed that COVID-19 statin users were of the older age group and strongly associated with comorbidities (hypertension, diabetes mellitus, coronary artery disease) and presented a higher respiratory signs and symptoms frequency.

The predominance of COVID-19 in the elderly can be attributed to the following factors: 1. aging immune system, 2. Inflammation and cytokine storms, 3. Epigenetic changes, and 4. Existing comorbidities. The immune system is the main defensive factor that suppresses and eliminates the SARS-CoV-2 from the host. During aging, the immunosenescence phenomena cause alteration in signaling and clearance pathways. At an older age, the alveolar macrophages fail to recognize the viral particles and cause excessive lung damage resulting in ARDS requiring mechanical ventilation. The epigenetic dysregulation, advanced biological aging, and host immune dysfunction trigger cytokine storms and pose a higher mortality risk [1, 3, 6, 7].

A high incidence of hypertension (75%) in our cohort study among hospitalized COVID-19 patients can be related to renin-angiotensin system (RAS) imbalance and endothelial dysfunction. Endothelial dysfunction occurs when the blood vessel wall becomes stiff and has diminished vasodilation. The persistent elevated systemic blood pressure causes premature aging and impairment of endothelium. The associated SARS-CoV-2 infection induces chronic inflammatory infiltration causing loss of homeostatic properties within the blood vessels [32]. The co-occurrence of NADH/NADPH oxidase in hypertensive COVID-19 patients accelerates the development of pulmonary damage-producing respiratory manifestations, as seen in our cohort study.

The importance of recognizing diabetes as a risk factor in COVID-19 is mainly related to the worse outcomes in such patients. Type 2 diabetes is characterized by a mild chronic inflammatory infiltrate due to higher visceral adipose tissue, diminished glucose regulation and homeostasis, and poor peripheral insulin sensitivity. The potential pathogenic mechanisms contributing to the predisposition of COVID-19 infection in diabetes patients can be due to decreased viral clearance, diminished T cell function, higher susceptibility to chronic inflammation, and cytokine storms [33, 34].

The predominance of the respiratory features is mainly attributed to increased inflammation and cytokine storms in aged patients [35]. The SARS-CoV-2 invades the alveolar epithelial cells through the ACE-2 receptor and undergoes replication. The virus releases numerous cytokines and interleukins, causing a cytokine storm [36–38]. The inflammatory markers cause the respiratory manifestations and as reflected by the laboratory markers in our study. The cytokine storm, if poorly controlled, leads to pneumocyte loss and diffuse alveolar damage, eventually progressing to ARDS [39].

Statins are 3-hydroxy-3-methyglutaryl coenzyme A (HMG-CoA) reductase inhibitors. Statins improve the endothelial integrity in patients with atherosclerosis and hypercholesterolemia by improving the eNOS mRNA causing upregulating the Rho/ROCK pathway. The other notable mechanisms of statin effects are Akt activation, attenuating cytokine-mediated vascular smooth muscle cells, and upregulation of Nitric oxide resulting in inhibition of adhesion molecules [40, 41]. The recent molecular study considered the role of statins in suppressing TLR4/MyD88/NF kB signaling pathways [42]. In cardiac complicated COVID-19 patients, the molecular role of intracellular molecule inflammasome NLRP3 is well described. The NLRP3 is activated through the oxidized LDL and TNF α, causing cytokine storms and cardiac complications in COVID-19 patients [43, 44]. Statins suppress and inhibit NLRP3 through reduced LDL oxidation and TNFα, causing a favorable lipid profile as indicated in our study.

Elevated levels of circulating D-dimer are known to be associated with an increased risk of developing thrombosis. In COVID-19 patients, the virus targets the endothelium resulting in loss of endothelial anti-thrombotic properties. The additional effect of COVID-19 associated endotheliitis affects the coronary and the cerebral blood vessels precipitating thrombus formation and leading to severe ischemic episodes [45, 46]. Statins cause a notable downregulation of coagulation cascade due to reduced tissue factor expression, reduced thrombin formation, and fibrinogen cleavage. Statins upregulate the endothelial function and antagonize the COVID-19 induced endothelial effects by attenuating VEGF and serum PAI-1 levels [47–49]. This positive effect of statins on the coagulation cascade is shown by lower circulating D-dimer levels, as observed in our study. The positive effects of statins in COVID-19 patients are also contributed to the anticoagulant property of statins. Statins inhibit NF-ҡB, reduce toll-like receptor 4 (TLR4), causing an overall anti-inflammatory state [50, 51]. The anti-inflammatory state is reflected by lower WBC count C- reactive protein levels, as seen in our study.

The circulating lipoprotein movement across the endothelium is mainly dependent on the transcytotic transport system. A lipoprotein smaller than 70 nm is the select size for transcytosis; however, in atherosclerotic patients, the triglyceride-rich lipoprotein carries a high percentage of cholesterol per particle, resulting in approximately 40 times larger than the normal lipoprotein [52]. This large molecule is arrested within the endothelium resulting in intimal plaques and promoting atherosclerosis and associated complications [53]. Our study reveals that even a moderate elevation of triglyceride can increase the risk of atherosclerosis significantly. The statistical analysis confirms that statins have a cholesterol-reducing effect by lowering the triglyceride and increasing the HDL levels in patients diagnosed with hypertriglyceridemia compared to patients without hypertriglyceridemia.

The beneficial role of statins in COVID-19 patients is well documented in the literature [10, 29, 54]. The recent implementation of statins as preventive and treatment options in COVID-19 patients has been approved by the National Health Institute in the USA [55]. Statins are considered safe, cost-effective, and beneficial in hospitalized COVID-19 patients due to their favorable clinical outcomes, as seen in our study [10].

Numerous studies have favored the use of statins in COVID-19 patients [10, 29, 54]. The National Health Institute in the USA has even recommended that COVID-19 patients continue statin therapy to prevent and treat cardiovascular disease [55]. This safe and cost-effective drug has been proven to be beneficial during hospitalization and linked with better clinical outcomes [10]. Statin users with cardiac diseases are known to have lower thromboembolic episodes due to the agonist action of statins on the coagulation cascade and its immune-inflammatory properties, thereby reducing the mortality rates in such cohort populations [51, 56]. In patients with acute severe COVID-19 illness, combined drug therapy using statins with angiotensin receptor blockers (ARB’s) is proven to be beneficial. Statins with ARB’s act on Angpt/TieZ and ACE2/angiotensin/Mas signaling within the endothelial and epithelial cells. The combined statin and ARB therapy have lower mortality in patients with influenza, Ebola virus, ARDS, and sepsis [57]. Grimalidi et al. discussed the negative effect of statins in patients with ARDS and sepsis [58]. The adverse effects of statins coupled with their pharmacokinetic effects in the liver by hepatic isoenzymes CYP3A4 are the main reasons for the physician’s reluctance in considering statins as a supplement therapy for COVID-19 patients [59]. These patients also require careful monitoring of creatine kinase and liver function tests during their hospitalization.

The literature studies providing insights on the protective role of statins in COVID-19 patients should be viewed with caution as most of the involved patients have pre-existing comorbidities, including diabetes, hypertension, cardiovascular and cerebrovascular diseases [10, 29, 31, 54]. The association of the comorbidities in COVID-19 patients can exacerbate the overhaul prognostic outcome [60]. The individual clinical factors act as confounders producing statistical variance and bias in any observational study [27]. The confounders need to be addressed by implementing multivariable propensity scores as done in our study. However, the current vaccination programs held in large scale magnitude have further benefitted the prognosis of statin users and COVID-19 patients [61].

In our study, analyses from three multispecialty hospitals during the COVID-19 pandemic have demonstrated that prior statin use significantly reduces the hospital mortality rate. However, to confirm these observational findings, a more rigorous randomized control trial would be authoritative to recommend the beneficial use of statins in COVID-19 patients in this country [62, 63].

### Strengths and limitations of this study

This study is a retrospective cohort study conducted in India, demonstrating the effects and outcome of statins in COVID-19 hospitalized patients. This study was conducted during the COVID-19 pandemic, and the results were statistically evaluated to support the beneficial effects of statins in COVID-19 patients. The limited literature studies on the effect of statins on hospitalized COVID-19 patients validate the results presented in our manuscript. A study done by Zhang et al. showed some variations in their results compared to our study; this is most likely due to the differences in the study size (< 10% of hospitalized patients were statin users) and the genetic form of the Chinese population [10]. Most studies originated from China, and a few recent meta-analyses showed the reflection of statin effects in the European and North American patient population. However, the findings and results varied greatly in study size, varying types, and dose of statin regimes [29, 64]. With the results in the previous observational studies and the significant inference from the present study, the results of the progressing clinical trials will be critical.

Cohort studies always attract statistical discordance in the form of confounding bias. In our study, this was limited by applying propensity scores and multivariable logistic regression analysis. The total number of cases in this cohort study was limited compared to India’s official COVID-19 case numbers. This can be explained due to the rigid inclusion and exclusion criteria followed in this study, which resulted in rejecting a high proportion of cases. The electronic data search further amplified the exclusion numbers as a lack of laboratory investigation added it to the exclusion category. The clinical findings, tracing statin treatment post-COVID-19 diagnosis, discontinuation of statins before hospitalization, and lack of data availability of patients with prescribed statins for preventive conditions were all contributory factors for lower numbers in our study.

## Conclusion

This study proposes that lipid-lowering drugs can benefit COVID-19 patients through their anti-inflammatory, anti-thrombotic, and pleiotropic effects on endothelial cells. The results elaborated in our study are clinically relevant and supported by previous observed cohort studies. Hence, the prospect of conducting a more rigorous randomized controlled trial in the future on a large number proportionate to the country’s regional population would provide more robust evidence on the beneficial effects of lipid-lowering drugs in COVID-19 patients and for clinical recommendation by practicing physicians and specialists.

### Summary of the study

- This study is a retrospective cohort study to be conducted in India demonstrating the protective role of statins in COVID-19 hospitalized patients
- This study was conducted during the COVID-19 pandemic, and the results were statistically evaluated to support the beneficial effects of statins in COVID-19 patients
- The findings in this study can be used for conducting more rigorous randomized control trials in India and globally.
- Cohort studies are usually associated with bias because of confounding factors. These factors have been minimized in our study by using propensity match scores
- This study provides a valuable conclusion and supports the beneficial effects of statins in COVID-19 patients
- The COVID-19 patients often presented with significant comorbidities that included hypertension, diabetes, obesity, and a previous episode of ischemic heart disease. This favors the use of statins in these patients
- Statins reduce the lipid levels and enhance the vascular endothelial function, causing a significant reduction in mortality due to coronary artery disease complications.
- Statins are safe, cost-effective, and proven to be beneficial during hospitalization and linked with better clinical outcomes.

## Data Availability

All data and codes generated for this study were de-identified and included in this manuscript. Data can be provided upon reasonable request to the corresponding author following approval by the Joint-Hospital Review Board.
